# Manipulating Autophagic Processes in Autoimmune Diseases: A Special Focus on Modulating Chaperone-Mediated Autophagy, an Emerging Therapeutic Target

**DOI:** 10.3389/fimmu.2015.00252

**Published:** 2015-05-19

**Authors:** Fengjuan Wang, Sylviane Muller

**Affiliations:** ^1^Immunopathology and Therapeutic Chemistry/Laboratory of Excellence MEDALIS, CNRS, Institut de Biologie Moléculaire et Cellulaire, Strasbourg, France; ^2^University of Strasbourg Institute for Advanced Study, Strasbourg, France

**Keywords:** chaperone-mediated autophagy, antigen MHCII presentation, systemic lupus erythematosus, autoimmune diseases, lysosomal dysfunction, P140 peptide/Lupuzor

## Abstract

Autophagy, a constitutive intracellular degradation pathway, displays essential role in the homeostasis of immune cells, antigen processing and presentation, and many other immune processes. Perturbation of autophagy has been shown to be related to several autoimmune syndromes, including systemic lupus erythematosus. Therefore, modulating autophagy processes appears most promising for therapy of such autoimmune diseases. Autophagy can be said non-selective or selective; it is classified into three main forms, namely macroautophagy, microautophagy, and chaperone-mediated autophagy (CMA), the former process being by far the most intensively investigated. The role of CMA remains largely underappreciated in autoimmune diseases, even though CMA has been claimed to play pivotal functions into major histocompatibility complex class II-mediated antigen processing and presentation. Therefore, hereby, we give a special focus on CMA as a therapeutic target in autoimmune diseases, based in particular on our most recent experimental results where a phosphopeptide modulates lupus disease by interacting with CMA regulators. We propose that specifically targeting lysosomes and lysosomal pathways, which are central in autophagy processes and seem to be altered in certain autoimmune diseases such as lupus, could be an innovative approach of efficient and personalized treatment.

## Introduction

Autophagy, an intracellular degradation pathway in which lysosomes play a central role, has been raised as a hot topic in almost every aspect of cellular processes, including immune responses and regulation. As it could be expected, therefore, any alteration of autophagy processes can potentially affect the normal course of cell metabolism and give rise to more or less dramatic cell malfunctioning. It is precisely what is more and more often emerging from experimental studies. Nowadays, there is growing evidence reporting the implication of autophagy alteration in a variety of pathological indications. In particular, some autophagy failures have been suggested or experimentally demonstrated in situation of chronic inflammation and autoimmune diseases.

Antigen presentation is a vital step in immune regulation where antigen-presenting cells (APCs) process antigens into short peptides, which are then loaded in to major histocompatibility complex (MHC) class I (MHCI) and II (MHCII) molecules and presented in this context to T cells. Classical (professional) APCs include dendritic cells (DCs), macrophages, Langerhans cells (LCs), and B cells. Beside this canonical antigen presentation pathway, DCs and non-APCs can acquire MHCI and/or MHCII molecules from neighboring cells through a pathway of cell–cell contact-dependent membrane transfer called trogocytosis. These so-called “MHC-dressed cells” subsequently activate or regulate T cells via peptide–MHC complexes without requiring any further processing (1). Exosome-mediated transfer might also contribute to this process.

Cells use a variety of mechanisms to generate antigens that will be presented in the context of MHC molecules to the receptor of T cells (TCR). Among these pathways, autophagy is considered a major process for the delivery of cytosolic and nuclear antigens to MHCII molecules in mature or late endosomes, also known as MIIC compartments (2–7). Abnormalities in antigen presentation have been proposed to play an important role in autoimmune diseases. In systemic lupus erythematosus (SLE), for example, hyperactivity of T cells resulting from panoply of factors might be related to abnormal antigen presentation by APCs (8, 9). Control of the autophagy pathway is thus critical, and pharmacological intervention targeting specific steps of this complex process could be determining at reprograming some dysfunction of the immune system occurring notably in inflammatory and autoimmune diseases (10–16).

Three major forms of autophagy have been found ubiquitously in eukaryotic cells, namely microautophagy, macroautophagy, and chaperone-mediated autophagy (CMA). Microautophagy is featured by a direct engulfment of cytosolic portions through lysosomal invagination. It is the least studied process among the three processes, yet microautophagy-like process has been shown to deliver cytosolic proteins to late endosomal compartments in DCs and might represent an important alternative route for antigen presentation (17, 18). This microautophagy-like degradation pathway does not depend on the canonical autophagy machinery and is distinct from CMA.

**Figure 1:**
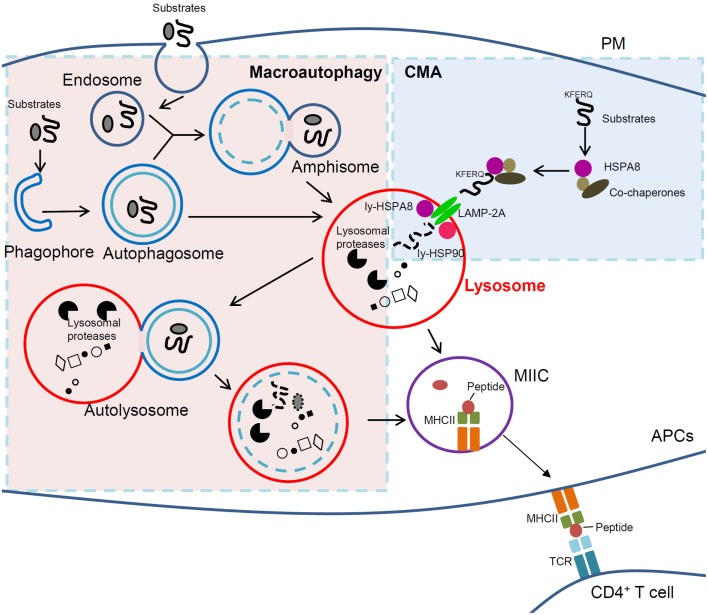
**Cross-talk between macroautophagy and CMA in immunity**. In macroautophagy (left, light pink region), amphisomes and autophagosomes that *contain elements of a cell’s own cytoplasm*, fuse with lysosomes to form autolysosomes in which autophagic substrates are degraded. In CMA (right, light blue region), however, cytosolic substrates that possess a *KFERQ-like motif* are recognized by HSPA8 and directly delivered to lysosomal receptor LAMP-2A together with other co-chaperones followed by degradation into lysosomes. In APCs, lysosomal compartments (lysosomes and autophagosomes) can further derive to MIIC. In this compartment, antigenic peptides are loaded into MHCII molecules, which can thus be delivered to the PM and recruit/activate CD4^+^ T cells. Clearly lysosomes are the central player for antigen presentation and immunity, and any dysfunction of lysosomal can lead to immune defects and to autoimmunity in particular. If selective, targeting lysosomal pathways (such as autophagic pathways) could thus become a strategy of choice to treat autoimmune patients. Macroautophagy and CMA are normally kept in a delicate balance and are intrinsically linked. Therefore, cautions need to be taken when intervening on one of the two pathways in autoimmune therapy, as the other could be potentially affected. APCs, antigen-presenting cells; CMA, chaperone-mediated autophagy; LAMP-2A, lysosomal-associated membrane protein type 2A; ly-HSPA8/ly-HSP90, lumenal (lysosomal) HSPA8 and HSP90; MHCII, major histocompatibility complex class II; MIIC, MHC class II compartment; PM, plasma membrane; TCR, T-cell receptor.

Macroautophagy is the most intensively studied form of autophagy and the vast majority of currently published data on the role of autophagy in antigen presentation result from investigation based on this basic autophagic process. In the macroautophagy pathway (Figure 1), double-membrane structures are generated to engulf whole cytosolic components, including organelles, and form autophagosomes. The latter then use microtubular tracks to encounter and fuse with lysosomes/late endosomes to form vesicles called autolysosomes where lumenal hydrolytic enzymes degrade cargo (microtubules have to be acetylated to allow fusion) (19). Initial description of macroautophagy process referred to random sequestration of cargo only. Nowadays, we know that macroautophagy can also occur in a more selective manner. Thus, mention may be made of aggrephagy (for aggregated proteins), mitophagy (mitochondria), ribophagy (ribosomes), pexophagy (peroxisomes), reticulophagy (endoplasmic reticulum, ER), lipophagy (lipid droplets), and xenophagy (pathogens), showing that in fact macroautophagy participates in a highly selective and tightly regulated process of substrate delivery (20–22). Direct evidence has been reported that endogenous antigen presentation depends on macroautophagy (3, 23–26), and activation of macroautophagy could facilitate presentation of intracellular peptide on MHCII molecules (2). In DCs used as APCs, the autophagy-related gene *(ATG)* 5, a key autophagy gene, seems to be required for antigen presentation (26). While mice with DC-conditional deletion in *Atg5* displayed no development defect, they showed, however, important failure mounting a normal T-cell response linked to improper processing and presentation of cytosolic antigens on MHCII molecules.

The third main type of autophagy, CMA (Figure 1), is a process where client proteins containing specific motifs related to KFERQ (present in about 30% of soluble cytosolic proteins) (27) are selectively recognized by the cytosolic chaperone protein HSPA8/HSC70 present in a co-chaperones-rich complex that delivers them to the lysosome membrane. Examples of such client proteins/substrates are glyceraldehyde 3-phosphate dehydrogenase, the E3 ubiquitin ligase ITCH, the calcineurin inhibitor RCAN1, the neuronal alpha-synuclein and tau proteins, galectin-3, and HSPA8 (28–30). The complex containing HSPA8 associated to the substrate then binds to the so-called lysosomal-associated membrane protein type 2A (LAMP-2A), acting as a monomer at this stage. LAMP-2A (but not LAMP-2B or LAMP-2C, or HSPA8) is exclusive for CMA. The binding of substrates to LAMP-2A leads to its multimerization, likely with the help of lysosomal HSP90 protein; the substrates undergo unfolding and reach the lysosome lumen through the LAMP-2A-enriched translocation complex with the aid of lysosomal HSPA8, where they are degraded by lysosomal proteases. LAMP-2A multimers then disassemble and degrade for the next cycle of CMA (28). This process, which is finely regulated (29), is carried out at basal level and can be activated under prolonged starvation and other stresses. It works then as an alternative energy sources and quality control to remove damaged proteins upon stress. CMA dysfunction (enhancement or slowdown) seems to directly or indirectly contribute in many diseases including neurodegenerative pathologies, metabolic diseases, and cancer (31–34). Recent evidences from our laboratory and others strongly support the importance of CMA in antigen presentation and pathological conditions, especially in autoimmunity.

The role of macroautophagy in antigen presentation has been described extensively in comprehensive reviews (7, 16, 35–41). Original data also accumulated supporting a central role for macroautophagy in both innate and adaptive immune responses, which greatly influence antigen presentation (42–45). Our intention herein is therefore to orientate our discussion on recent findings supporting the prominent role of autophagy in antigen processing and presentation, with a particular focus on CMA and autoimmunity. A special emphasis will be given on lupus, as several recent studies have provided novel results that shed new light on this syndrome and may have important therapeutic issues. The topic of this review will also address in lupus the possible inherent dysfunction of lysosomes, a central organelle that has been seldom examined in autoimmune settings.

## CMA and Antigen Presentation

While the implication of macroautophagy in antigen presentation has been extensively investigated in independent laboratories, that of CMA in this process has seldom been studied. Experimental data supporting the view of a direct implication of CMA in antigen presentation have been provided by Blum and collaborators who demonstrated that by manipulating the expression of LAMP-2A or HSPA8 (the two key components of the CMA pathway) in stable human B-cell line, enhanced or reduced cytoplasmic autoantigen presentation could be observed (4). Silencing total cellular LAMP-2 levels reduced the MHCII presentation of the endogenous antigen glutamate decarboxylase (GAD) presented in the context of HLA-DR4 (presentation of exogenous antigens, such as human serum albumin, was also decreased in cells with reduced LAMP-2A and LAMP-2B levels). Overexpression of LAMP-2A isoform led to an increased presentation of endogenous GAD but not of exogenous antigens. Overexpression of LAMP-2B, however, had no effect on cytoplasmic or extracellular antigens. Similar observations were made using cells in which the levels of HSPA8 expression were modified. These data suggest a role for CMA in endogenous MHCII antigen presentation. Further experiments using a set of endogenous antigens corresponding or not to CMA substrates should consolidate these findings.

It is not only in antigen presentation that CMA plays important role, but also in T-cell regulation. Recent data have revealed that CMA is an essential regulatory element of T-cell activation through the targeted degradation of negative regulators of TCR signaling, namely the ubiquitin ligase Itch and the calcineurin inhibitor RCAN1 (30). This study provides the first evidences that TCR engagement induces CMA, likely through the generation of reactive oxygen species (ROS), a CMA inducer. Deletion of LAMP-2A gene abolishes *in vivo* T-cell response to immunization or infection.

## CMA Alteration in Autoimmunity

In immunity, autophagy has emerged some years ago as a process, the deregulation of which could lead to a breakdown of tolerance to self and autoimmunity [reviewed in Ref. (16, 46–51)]. Following the discovery that several *ATGs* could be associated to autoimmune syndromes, e.g., *ATG5*, PR domain zinc finger protein 1 (*PRDM1*; also known as *BLIMP-1*), DNA-damage regulated autophagy modulator 1 (*DRAM1*) in SLE patients (52–56), or *ATG16L1* and immunity-related GTPase M (*IRGM*) in Crohn’s disease and ulcerative colitis (57–61), further studies, at the cellular and molecular levels, were undertaken. Alterations of macroautophagy were effectively found to occur in several immune cell subtypes from patients and model mice with lupus, in T cells (62, 63) and later on in B cells (64) and macrophages (65). Macroautophagy defects were also observed in rheumatoid arthritis (RA) (66, 67), multiple sclerosis (MS) (68), and type-I diabetes (69, 70), for example [reviewed in Ref. (71)].

Although most of these observations would require deeper investigations to reinforce the molecular and cellular mechanisms that underline the observed autophagy failures, the link existing between inflammatory autoimmune diseases and macroautophagy seems to be well established. It is far to be the case with CMA. Nowadays, the effect of CMA activity on human autoimmune diseases remains largely unknown. This lack of information likely results from the fact that murine models which display a genetic background of autoimmunity and that are further deleted for CMA markers LAMP-2A and HSPA8 could not be generated until now and that CMA-specific inhibitors are not available. Known data were obtained using a spontaneous murine model of autoimmunity, the MRL/lpr mouse that develops a strong lupus-like disease characterized by lymphadenopathy due to an accumulation of double negative CD4^−^CD8^−^CD3^+^B220^+^ T cells (72, 73). These mice (both females and males) display an accelerated mortality rate and produce lupus-specific autoantibodies [reacting with double stranded (ds) DNA, nucleosome, and ribonucleoprotein (RNP) Sm antigens]. In addition, they possess large amounts of immune complexes and cryoglobulins in their serum as well as, rather typical of RA, high incidence and titer of rheumatoid factors. The accelerated lupus-like phenotype observed in MRL/lpr results from a recessive autosomal mutation called lymphoproliferation (*Lpr*) that alters transcription of the FAS receptor (74).

MRL/lpr mice present many immune defects attributed to Fas deficiency, notably in CD4^+^ T cells and B cells. In addition to these defects that have been extensively investigated over years, we showed recently that the CMA pathway is deregulated. Both LAMP-2A and HSPA8 CMA markers were found to be over-expressed in splenic MRL/lpr B cells (75, 76), suggesting strongly that in this setting, there is a change in CMA activity. At this stage, it is not known if this alteration affects all or a subset only of lysosomes in MRL/lpr B cells and if other APCs are also affected. The molecular mechanisms that are implicated in these changes of expression are not known either but seem to be different. Whereas HSPA8 expression correlates with increased mRNA expression in MRL/lpr splenocytes, variation in mRNA were not observed for LAMP-2A (75, 76). In any event, the results that emerge from these two studies are pivotal since as detailed above, CMA may contribute to antigen presentation, and in the current setting the amount and/or the diversity and/or the nature of peptides that are loaded to MHCII molecules and presented to autoreactive CD4 T cells could be largely affected.

## Cross-Talk between Macroautophagy and CMA in Autoimmunity

Although macroautophagy and CMA are clearly distinct pathways with their proper regulation systems, they are closely linked *de facto* at the late fusion stages when the autolysosomes are formed from amphisomes and lysosomes or from autophagosomes and lysosomes (Figure 1). A cross-talk occurs between macroautophagy and CMA during starvation (77, 78). Macroautophagy is activated shortly after starvation and reaches its maximum level around 4–6 h. When CMA is stimulated (gradually after 8–10 h of starvation and for up to 3 days) (77), macroautophagy is first induced and then declines. It has also been demonstrated in these studies that inhibiting CMA by depletion of LAMP-2A induces macroautophagy and *vice versa* that blockage of macroautophagy via genetic and pharmacological approaches could activate CMA. On a functional point of view, there is clearly a kinetic and temporal balance between the two processes (79). This important aspect has to be taken into account in the development of molecules designed to specifically regulate one particular pathway.

## Lysosomal Dysfunction in Autoimmune Diseases

Endo-lysosomal compartments play vital roles in immune regulation. Their functions include trafficking, maturation of MHCII complexes, antigen processing and presentation, and signal transduction (80). The biological functions of lysosomes are mediated by various enzymes (glucosidases, proteases, and sulfatases) that are contained in their lumen and constituents of lysosomal membrane. The physiological environment that endosomes and lysosomes encounter (pH, amino acids concentration, ROS, Ca^2+^ content, lipid composition, and membrane potential) is essential for their proper functioning. A number of adverse elements can affect them, potentially leading to abnormal antigen presentation and altered immune responses (80–82). The central roles of lysosomes in immunity and autoimmunity are discussed below.

### The early hypothesis on “lysosomal fragility” in autoimmune diseases

The first hypothesis linking lysosomes and autoimmune phenomena was proposed by Weissmann and Thomas as early as in 1962. They proposed that “lysosome fragility” could be related to SLE following a rational reflection on two phenomena, namely (a) that ultraviolet light can exacerbate SLE manifestation in patients and (b) that lysosomes are highly sensitive to ultraviolet light both *in vitro* and *in vivo* (83). Although it was a genuine idea, this avenue has not been pursued. It is much later only, that experimental investigations revisited this hypothesis leading to the suggestion that lysosomal enzymes that are released from “fragile” lysosomes might be autoantigens in SLE (84). Detailed studies on the fragility of lysosomes in SLE and other autoimmune diseases are still relatively scarce and these assumptions remain to be experimentally consolidated. Current studies whose task is to link lysosomal dysfunctions and autoimmune diseases mainly focus on the activities of lysosomal proteases and lysosomal-related pathways in these indications.

### Lysosomal proteases in antigen presentation and autoimmunity

Antigen presentation relies on the lysosomal proteases. Numerous studies have been carried out to identify the specific proteases that are involved in the MHCII antigen presentation pathways (85–88). Lysosomal proteases include cysteine cathepsins families (B, C, F, H, K, L, O, S, V, W, and X), aspartyl cathepsins (D and E), and serine cathepsins (A and G) as well as asparagine endopeptidase (AEP). They are synthesized under the form of zymogens in the ER and transported to the Golgi apparatus where they are modified by the addition of mannose-6-phosphate. This tag facilitates their transportation to lysosomes where they get activated by the low-pH endo-lysosomal environment. Lysosomal proteases exhibit various functions in immunity, such as processing of the invariant chain (Ii), which is required for the maturation of MHCII complexes, proteolytic processing of antigen, and TLR-receptor signaling (88, 89).

The maturation of MHCII complexes is dependent on the processing of invariant chain Ii to Class II associated invariant chain peptide (CLIP-fragment). The invariant chain Ii is first degraded into a 22-kDa leupeptin-induced protein (LIP) intermediate, then to a 10-kDa small-leupeptin-induced protein (SLIP) intermediate and finally to the CLIP-fragment. The rate-limiting step is the final processing from SLIP to CLIP, which has been identified to be dependent on cysteine protease-cathepsin S. Inhibition of cathepsin S by either genetic knock-out or chemical inhibitors leads to accumulation of SLIP intermediates in B cells, macrophages, DCs, and T cells that can also express functional MHCII molecules (90, 91). Cathepsin L (92), V (93), and F (94) have been suggested to be also involved in this multistep process.

As the executors of antigen processing, lysosomal proteases represent “double-edged swords,” as they can either generate antigenic epitopes (in favor of their presentation) or destroy some epitopes (acting therefore against their further presentation). Lysosomal proteolysis is often assumed to favor production of ligands for MHCII molecules. Blocking lysosomal function and abolishing activity of lysosomal proteases by lysosome alkalization, for example, decrease antigen presentation (95–98). Mice deficient in mannose 6-phosphate, the tag that targets newly synthesized lysosomal proteases from ER to lysosomes (see above), show significant loss of various lysosomal proteases in B cells. This deficiency in lysosomal proteases leads to impaired antigen processing and presentation (99). In most cases, the processing of endocytosed antigens is not specific, as the cleavage site is determined mainly by its accessibility to the active site of proteases. Nevertheless, some studies have also shown that presentation of a particular epitope requires a certain type of lysosomal proteases, such as the essential role of AEP for tetanus toxin processing in human APCs (100).

Intriguingly in the case where lysosomal proteases play a destructive role for the antigen epitopes, limited lysosomal proteolysis still favors their presentation. Delamarre et al. demonstrated that bovine pancreatic ribonuclease RNase A is more immunogenic than its variant RNase S, which is structurally and enzymatically identical to RNase A, but more susceptible to lysosomal proteolysis (101). This suggests that in some cases, reducing the susceptibility of antigens to lysosomal proteolysis can nevertheless enhance their immunogenicity. It is noteworthy that macrophages are rich in lysosomal proteases capable of rapidly destroying internalized antigens whereas DCs and B lymphocytes, in comparison, are poorer in proteases, which makes them rather in charge of antigen processing and presentation (102). It is not clear whether this limited proteolysis is specific to certain proteases/antigens. Absence of one protease can also increase the presentation of certain epitope, as shown in the case of myelin basic proteins and myoglobin, which are readily presented by human APCs that lack AEP, and by mouse APCs that lack cathepsin D (103, 104). The dual role of lysosomal proteases in antigen presentation is therefore complex and still requires further elucidation. This question remains central as the final levels of the presented peptides (together with an array of co-stimulatory signals) determine the ultimate immune response, namely tolerance or autoimmunity.

Most importantly also, lysosomal proteases are known activators of endo-lysosomal pattern sensing receptors TLRs. Thus, endo-lysosomal proteases are found to activate TLR9 through the cleavage of its N-terminal region, which transforms the receptor to its signaling-active form. TLR3 and TLR7 have been shown to be activated similarly (105–107). Chemical inhibition and gene knock-out studies have indicated that AEP and cathepsin K are specific for TLR processing (108, 109).

Due to the central roles of lysosomal proteases in antigen presentation, maintenance of their activities is essential for immune responses. Perturbation of their activities has been found to be related to autoimmune diseases. For example, the expression levels and activities of cathepsins S and H have been shown to be increased in lacrimal glands of mouse models of Sjögren’s syndrome (SS) (110); tear cathepsin S has been further characterized in SS and proposed as a candidate biomarker for SS (111). Cathepsin D-like activity has been shown to be raised in the SLE patients’ serum, and at a lower extend in the serum of patients with progressive systemic sclerosis, chronic glomerulonephritis, and glomerulonephritis with nephrosis syndrome (112). Studies dealing with the expression levels and activities of lysosomal proteases still remain limited. They might have important application as it has been shown, for example, that cathepsin S inhibition could suppress lupus nephritis and other signs of the lupus disease (113). Omics studies would be needed to further identify deregulated expression of lysosomal proteases in autoimmune diseases.

### Lysosomal pH is essential for immune regulation

The maintenance of proper lysosomal functions is not only related to expression levels of lysosomal proteases, but also to their proteolytic activities, which largely depend on lysosomal pH environment. Most lysosomal proteases show their optimal activity in the acidic environment in lysosomes (pH 4.0–5.0). The acidification of lysosomes relies on the vacuolar H^+^-ATPase, a transmembrane multimeric protein complex, which pumps protons from the cytosol into the lysosomal lumen against their electro-chemical gradient by using energy from ATP hydrolysis (114, 115). Alteration of lysosomal pH, particularly lysosomal alkalization, was shown to contribute to the pathologies in several chronic diseases (116, 117).

**Figure 2:**
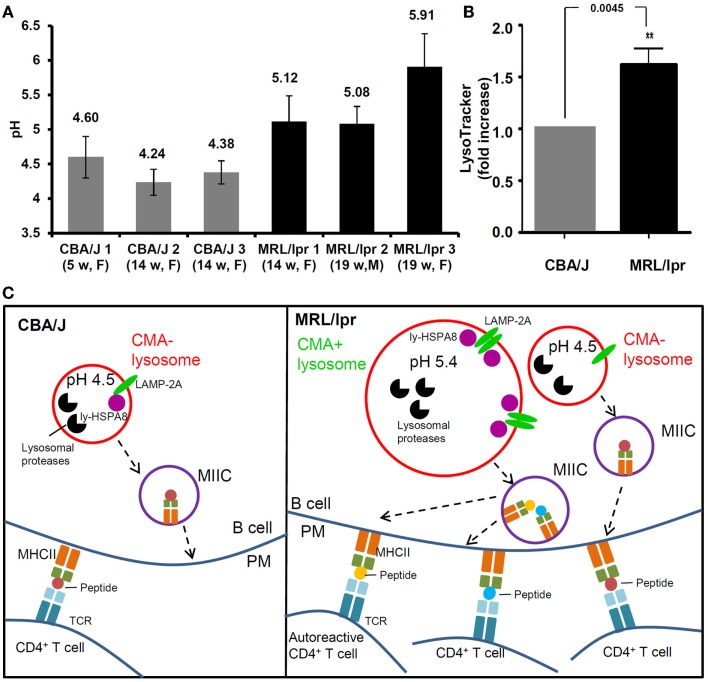
**Alteration of lysosomal functions in autoimmune diseases**. **(A)** The pH values of lysosomes are compared in the splenocytes (mainly B cells) of three healthy CBA/J mice and three MRL/lpr lupus-prone mice. Splenocytes were stained with LysoSensor Yellow/Blue DND-160, followed by ratiometric measurement at excitation wavelength 360 nm and emission wavelengths both 460 nm (blue) and 535 nm (yellow). The blue/yellow ratio was calculated after subtracting the background fluorescence. A standard curve of blue/yellow ratio versus pH was performed meanwhile in Raji cells by clamping their lysosomal pH in buffers with a series of pH and ionophores. The pH values of lysosomes were calculated from the standard curves. The error bars are standard deviations from three replicates. **(B)** The total volumes of lysosomal compartments are compared in the splenocytes from CBA/J and MRL/lpr mice (four mice of each strain) by LysoTracker Green staining DND-26 and flow cytometry measurement. The fold change of LysoTracker Green mean fluorescence intensity is plotted in the graph [modified from Ref. (75)]. **(C)** Schematic illustration of the differences between lysosomal functions in CBA/J and MRL/lpr B cells and the impact in autoimmunity (hypothetical scenario). In CBA/J mice and basal conditions, most lysosomes in B cells are CMA^−^; they show physiological pH (namely ~4.5) and a basal content of LAMP-2A and ly-HSPA8 CMA markers. In contrast, in MRL/lpr B cells, beside lysosomes that are not affected and remain mostly CMA^−^, subsets of lysosomes exhibit higher pH values (>5.0); in those B cells, the volume of lysosomal compartments is higher in average, likely due to increase in their sizes and/or numbers and the expression levels of lysosomal LAMP-2A and HSPA8 are elevated, indicative of higher CMA activity (CMA^+^ lysosomes). The alterations of lysosomes and CMA activity might both lead to higher antigen presentation at the B-cell surface (B cells serve as efficient APCs in lupus) and consequently hyperactivity of autoreactive CD4^+^ T cells. CMA, chaperone-mediated autophagy; CMA^+^/CMA^−^, CMA-active/-inactive lysosomes; F, female; LAMP-2A, lysosomal-associated membrane protein type 2A; ly-HSPA8, lumenal (lysosomal) HSPA8; M, males; MIIC, major histocompatibility complex class II compartment; PM, plasma membrane; TCR, T-cell receptor; w, weeks.

With a ratiometric fluorescent dye LysoSensor Yellow/Blue that is specifically designed for lysosomal pH measurement (118), we recently demonstrated that in the spleen, MRL/lpr B cells exhibit considerably higher pH than healthy CBA/J mice B cells, with mean lysosomal pH values of 5.4 and 4.3, respectively (Figure 2A) (76). This raised pH that can reach values close to 6 in some MRL/lpr mice, might lead to a decreased activity of lysosomal proteases. As discussed above, limited antigen processing can possibly result into enhanced immunogenicity (101). Altogether, these observations might explain at least in part the over-reactivity of peptide-reactive T cells to unique peptides presented by APCs in MRL/lpr mice (78, 119). Moreover, the increase of lysosomal pH in lupus B cells could affect the CMA process. The stability of lumenal HSPA8 is effectively superior when pH values are around 5.2–5.5 (78). This might explain in part the hyperactivity of CMA recently demonstrated in MRL/lpr B cells (76). At this stage, much more investigation would be required to consolidate the link that may occur between a higher lysosomal pH and a hyperactivity of B cells in MRL/lpr mice. Likely due to the lack of convenient and highly effective probes for lysosomal pH measurement, few data are available in the literature in terms of perturbation of lysosomal pH in autoimmune diseases. Our pH measurement in the MRL/lpr mice model suggests that increase of lysosomal pH is an important aspect of lysosomal malfunction that needs to be evaluated in SLE and other autoimmune diseases.

Beside the increased lysosomal pH, an augmentation of total volume of acidic compartments was also observed in B cells from MRL/lpr mice (Figure 2B) (76). This result was obtained following staining of splenocytes by LysoTracker Green and FACS analysis. A similar observation was published using MRL/lpr liver Kupffer cells (120). This finding might be related to an increased size of lysosomes and/or an increase of lysosomal number, which needs to be further investigated. One can hypothesize that lysosomal biogenesis could be altered in autoimmune diseases leading to hyperactivity (quantitative or qualitative/selective) of antigen presentation in APCs that can deregulate the responsiveness of autoreactive CD4^+^ T cells (Figure 2C).

### Lysosomal storage diseases and autoimmune responses

Lysosomal storage diseases (LSD) diseases are genetically inherited metabolic disorders due to defects in lysosomal enzymes that are essential for the metabolism of proteins, lipids, and other cell components. The accumulation of non-degraded materials inside lysosomes can lead to their dysfunction. Some immune system abnormalities, either suppression or hyperactivity, associated with LSDs have been described (121, 122). Hyper-responsiveness of immune cells was notably observed in LSDs such as mucopolysaccharidosis IIIB (MPS IIIB), GM2 gangliosidosis, globoid cell leukodystrophy, Niemann-Pick type C1, and juvenile neuronal ceroid lipofuscinosis [reviewed in Ref. (121)]. In MPS IIIB, a pathogenic autoimmune response directed to central nervous system (CNS), components independent of LSD pathology were identified (123). MPS IIIB is characterized by autosomal recessive defects in α-*N*-acetylglucosaminidase, a lysosomal enzyme, which degrades heparin sulfate (HS), a glycosaminoglycan (GAG), which is important for biological homeostasis (it is known in particular to modulate the sensitivity of T cells and APCs upon stimulation). Aberrant GAG metabolism has been described to be associated to several autoimmune diseases, such as RA, scleroderma, and SLE (124–126). Consistently in their MPSIIIB mouse model, Killedar et al. observed enhanced autoimmune CNS antigen presentation (123). Although a direct link between LSDs and autoimmune diseases is not clearly established, it is tempting to take into account the central role of lysosomes in autoimmunity when considering LSDs.

It should be added that a number of studies have highlighted some defects in the autophagy-lysosome process in LSDs and relevant murine models (122, 127–130) with notably, some failure in the autophagosome clearance. These features led Lieberman and colleagues to conclude that LSDs could be seen primarily as “autophagy disorders” (127).

## Therapeutic Molecules That Target Autophagy and Lysosomes

Due to the essential role of lysosomes in immunity, modulating lysosomal functions has been put forward as a right therapeutic strategy for inflammatory diseases (16, 131, 132). An established way of lysosomal intervention is through inhibition of lysosomal acidification. In their pioneering work published more than three decades ago, Ziegler and Unanue demonstrated the inhibitory effect of chloroquine (CQ) on antigen presentation to T cells (133). In the context of autoimmune indications, mention should thus be made of drugs that increase lysosomal pH such as CQ and hydroxychloroquine (HCQ; plaquenil™), which have been shown to be effective in SLE (134–137), SS (138), RA (139, 140), and a rat model of MS, the experimental autoimmune encephalomyelitis (141). Note that nowadays, the mechanism of action of CQ/HCQ is still a matter of debates. Any direct effect of CQ/HCQ on CMA process was not described and it is not known if these molecules play an effect on CMA-active and/or -inactive lysosomes. Data are also lacking regarding their possible effect on the lysosomal pH of various immune cells when used at pharmacological concentration range. It is indeed perplexing whether and how CQ/HCQ could further enhance the lysosomal pH in the splenocytes (primarily B cells) of MRL/lpr mice that already exhibit a higher lysosomal pH than that measured in CBA/J splenocytes. It is even unclear if efficacy of CQ and HCQ in SLE, SS, and RA results solely from their ability to increase the lysosomal pH, as this family of molecules exerts very diverse, possibly indirect, effects (142). The lack of selectivity of a drug generally originates undesirable secondary effects. CQ is effectively known to exhibit retinal toxicity. Serious vision loss that is usually irreversible has been observed. Although HCQ generates much less side effects, the risk of HCQ toxicity rises to nearly 1% with a total cumulative dose of 1.0 g, which corresponds to ∼5–7 years of normal use. Annual ophthalmologic examinations are therefore recommended 5 years onward under HCQ therapy, and in some cases before, if there are known risk factors (143). A number of HCQ analogs and mimics have been tentatively designed that keep the molecule activity without adverse effects. Ongoing research should provide such safe molecules in the future.

Besides CQ and HCQ, other alkalinizing lysosomotropic drugs that affect lysosome function have been described. They are amodiaquine (Camoquin™, Flavoquine™), a 4-aminoquinoline compound related to CQ, and azithromycin (Zithromax™, Azyth™, or Azithromycin™), for example. The latter, a potent macrolide antibiotic, is notably used for treating chronic inflammatory lung diseases, such as cystic fibrosis (CF). Long-lasting use of azithromycin by CF patients can lead to deleterious effects, notably some infections by non-tuberculous mycobacteria. It was found that azithromycin, which affects lysosome acidification, provokes a blockade of autophagosome clearance and a much weaker intracellular killing of mycobacteria (144). The MF6p/FhHDM-1 major antigen secreted by the trematode parasite *Fasciola hepatica* has also been reported to suppress antigen processing and presentation selectively in macrophages by inhibiting vacuolar ATPase activity, which consequently reduces lysosomal acidification (132).

The second approach to modulate lysosomal function is by using protease inhibitors. Inhibition of lysosomal acidification (such as by HCQ) can inhibit lysosomal protease activity, though without selectivity. Some inhibitors specific for individual lysosomal proteases were developed and might have some promise for therapy of autoimmune diseases. Due to its key role in the maturation of MHCII complexes, together with the fact that its expression and/or activity has been shown to be increased in some autoimmune diseases (see above), cathepsin S, in particular, represents an attractive target for such therapeutic options (88, 145). The orally available cathepsin S inhibitor RO546111 was shown to reduce the activation of splenic DCs and the subsequent activation of CD4^+^ T cells, to diminish hypergammaglobulinemia and anti-dsDNA antibody levels and prevent lupus nephritis progression in MRL/lpr lupus mice (113). Cathepsin S inhibitor Clik 60, a molecule developed with the help of computer-graphic modeling method based on the stereo-structure, shows suppression of autoimmunity in a murine model for SS (146). New classes of cathepsin S selective inhibitors have been developed (88, 147), and some of them, such as RWJ-445380 and CRA-028129, are currently evaluated in late stage clinical trials for the treatment of RA and psoriasis. The activity of cathepsin K inhibitors, such as blicatib, relacatib, and odanacatib, was also investigated (88). In a phase-III clinical trial, it was observed a significant reduction of fractures in post-menopausal women under odanacatib (148). Treatment of CA-074, a cathepsin B inhibitor, was also reported to suppress autoimmune responses in a mercury-induced autoimmune mouse model (149). Targeting endo-lysosomal proteases remains, however, a difficult pharmacological task due to the fact that single proteases can have multiple functions (88). Furthermore, there is a cross-talk between proteases, and inhibiting one specific protease can up-regulate or down-regulate another one. Many more studies are therefore required before pharmacological interventions based on this strategy can be generalized without risk.

The third strategy to target lysosomes is through modulation of autophagic pathways. Several drugs used experimentally or currently under preclinical trials for SLE, such as glucocorticoids, HCQ, rapamycin, P140 (Lupuzor™), bortezomib (Velcade™; the first approved proteasome inhibitor), vitamin D, and cyclosporine A have been found, sometimes incidentally and belatedly, to act as potent activators or inhibitors of autophagy processes (16). The very large majority of these molecules interfere with macroautophagy and in general they also act on other, non-autophagic targets (10–16, 51, 150). Since, as detailed above, macroautophagy and CMA are maintained in a finely regulated balance (this is also the case between autophagy and apoptosis or autophagy and the proteasomal system, for example), discontinuing one process could lead to abnormality of the other. Thus, again, along with other strategies described above, pharmacological interventions targeting autophagy pathways require advanced mechanistic investigations to be made.

## The Phosphopeptide P140, a Potent Immunomodulator of the T-Cell Response in SLE

Among the numerous molecules claimed to activate or inhibit autophagy, very few in fact primarily target the CMA pathway (151, 152). Noteworthy also is the recognition that some small molecules initially described as specific CMA modulators have since proven to exhibit other activities, which somewhat diminishes their initial interest (29, 153). In this context, the remarkable properties of the so-called peptide P140 must be highlighted. This peptide, which significantly reduces the biological and clinical defaults of SLE in patients and delay mortality of treated MRL/lpr mice, binds and co-localizes *in vivo* with HSPA8, a major chaperone of the CMA and macroautophagy processes (154). P140 is a 21-mer linear peptide (sequence 131–151) derived from the small nuclear RNP U1-70K. A phosphoserine residue was introduced at position 140 during its chemical synthesis (hence its name). In a multicenter, randomized, placebo-controlled phase IIb study, P140/Lupuzor was safe and met its primary efficacy end points in lupus patients (155). These results confirm previous data generated in MRL/lpr lupus-prone mice in which the preclinical studies were performed (156, 157) and those obtained in an open multicenter phase IIa clinical study in which 20 lupus patients were enrolled (158). A phase-III clinical trial will start shortly in North America and West Europe.

Recently, the pathway taken by the peptide P140 intracellularly and its potential mode of action have been elucidated (76). In MRL/lpr B cells, the phosphorylated peptide P140, but intriguingly not the non-protective unphosphorylated peptide, uses the clathrin-dependent endocytosis pathway to reach and accumulate into lysosomes. Based on *in vitro* data, it is assumed that in the lysosomal lumen, P140 compromises CMA, at least in part, by disruption of the lysosomal lumenal HSPA8 heterocomplexes containing HSP90. The consequence of this inhibitory effect on CMA may be a slowing-down or a qualitative change of cellular autoantigen loading to MHCII molecules and as a result, a weaker priming of autoreactive T cells. This effect potentially ends up by a beneficial reduction of autoreactive B-cell proliferation and differentiation into deleterious autoantibody-secreting plasma cells, leading to an improvement of the autoimmune status observed in patients with SLE (76).

**Figure 3:**
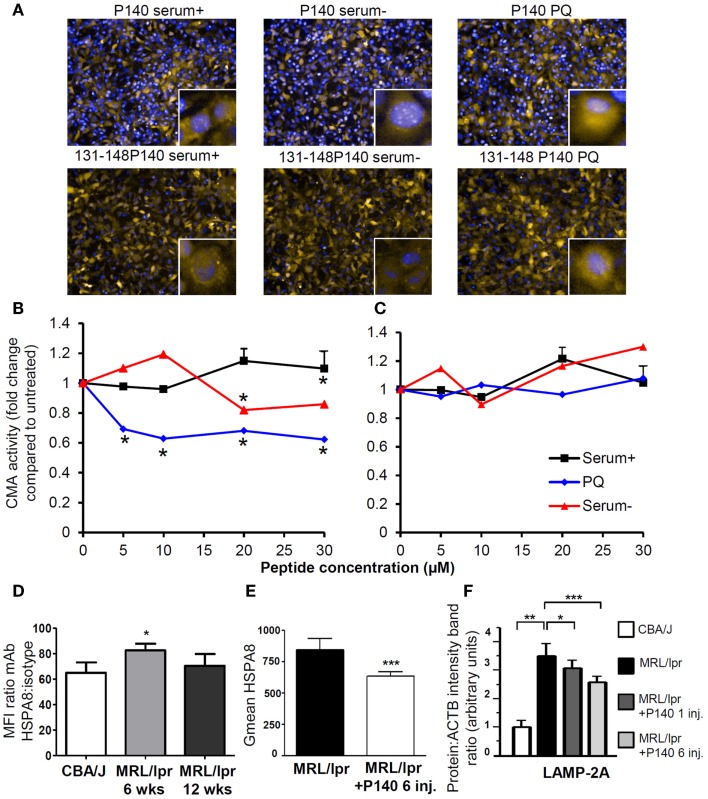
**P140 peptide, an immunomodulator that acts by inhibiting the CMA pathway**. **(A–C)** P140 peptide (30 μM) but not the truncated 131–148 P140 peptide analog (30 μM) used as control decreases CMA activity in a dose-dependent fashion. NIH3T3 cells stably expressing the photoswitchable CMA reporter KFERQ-PS-Dendra were photoswitched and maintained in serum-supplemented media (CMA^−^) or serum-supplemented media with PQ (CMA^+^), which induces CMA by generating mild oxidative stress. Cells were further exposed to P140 or control peptide 131–148 P140 at the indicated concentrations for 12 h. The fluorescence of CMA reporter was measured with high content analysis and representative images are shown in **(A)** [yellow = KFERQ-PS-Dendra; blue = DAPI]. The quantification was done by calculating the average number of KFERQ-PS-Dendra puncta in nine different fields (approximately 700 cells per condition) and divided by untreated control as the fold change of CMA activity and shown in **(B)** for cells treated with P140 and **(C)** with 131–148P140 for indicated concentrations. **(D)** The surface expression of HSPA8 was measured in B cells of 6- and 12-week-old CBA/J and MRL/lpr by flow cytometry. The fold changes were plotted by dividing the mean fluorescent intensity values of HSPA8 staining by those of isotype staining. **(E)** The surface expression of HSPA8 was measured in B cells of untreated MRL/lpr mice or of MRL/lpr that received six intravenous injections of peptide (100 μg P140/mice/injection) at daily intervals. The arbitrary geometric mean (G mean) values of HSPA8 fluorescence were plotted for MRL/lpr B cells with or without P140 injection. The error bars are standard deviation from three replicates. **(F)** The expression levels of LAMP-2A in purified B cells from CBA/J mice, untreated MRL/lpr mice (11–13-week-old), MRL/lpr mice that received one injection (and cells were collected 5 days later) or six injections (100 μg/mice/injection) of P140 peptide at daily intervals, were assessed by western blot and quantified by densitometry. ACTB, actin beta; CMA, chaperone-mediated autophagy; CMA^+^/CMA^−^, CMA-active/-inactive lysosomes; DAPI, 4′,6′-diamidino-2-phenylindole; inj., injections; LAMP-2A, lysosomal-associated membrane protein type 2A; PQ, Paraquat; wks, weeks.

This mechanism of action has been substantiated by *in vitro* experiments showing a direct effect of P140 on the CMA pathway (Figures 3A–C) in NIH3T3 cell line. It is also supported by *in vivo* data demonstrating that upon treatment of MRL/lpr mice with the P140 peptide, the levels of two key CMA components, namely LAMP-2A and HSPA8, which are over-expressed in lupus mice are significantly decreased in B cells of treated mice (Figures 3D–F) (75, 76). Other lysosomal proteins, such as cathepsin L and to a lower extent LAMP-1, follow the same trend. It should be noted that the mechanisms that modulate HSPA8 and LAMP-2A changes are different. Whereas HSPA8 protein expression correlates with its increased mRNA expression in MRL/lpr splenocytes (75), variations in mRNA were not observed for *Lamp-2a* (76). It is therefore possible that a modification in the stability of LAMP-2A occurs in the lupus model, and since HSP90 is involved in the stabilization of LAMP-2A (159), the weaker stability of LAMP-2A could in fact result from the effect of P140 with the HSPA8 heterocomplex containing HSP90. Among the other experimental data that reinforce the proposed mechanism are the findings that upon treatment of MRL/lpr mice with P140 peptide, there is a decreased expression of MHCII molecules (75), a much weaker reactivity of peripheral T cells toward peptides known to encompass T-cell autoepitopes (160), and lower levels of anti-dsDNA antibodies that are markers of the lupus disease (156). Most interestingly, compared to MRL/lpr mice that received saline, P140-treated MRL/lpr mice normally responded to a viral challenge and mounted specific CD4^+^ T-cell and antibody responses of equal magnitude (160). On a clinical point of view, dermatitis and renal damages were found to be diminished in MRL/lpr mice that received P140 (75). The data described above, obtained in a strong model of murine lupus and in patients with SLE, emphasize the effectiveness potential of a unique peptide that targets CMA and defective lysosomes to immunoregulate autoimmune responses. We trust that this kind of strategy may be harnessed in other pathological conditions in which reduction of CMA activity would be desired.

## Conclusion

Manipulating MHCII antigen presentation of antigenic fragments acquired via the endo-lysosomal pathway certainly represents an efficient strategy to immunomodulate and selectively deviate the autoimmune response. The development of this approach relies on a good knowledge of early processes subsequently leading to autoantigen processing, loading, and presentation by MHCII molecules. Greater understandings of the biogenesis and regulation of lysosomes and late endosomal MHCII compartment (MIIC), of their content in normal and variable pathological environments using advanced proteomics approaches (nowadays a technically unsolved challenge in the case of primary B lymphocytes), and of the role played by post-translational/epigenetic changes that can dramatically affect antigen degradation, are a few of some important research issues that deserve particular attention in the future. The discovery of novel mechanisms that control the lysosomal-autophagic pathways, notably in CMA, and their possible deregulation in pathological settings are pivotal in this frame. The P140/Lupuzor example demonstrates the feasibility to control an extraordinary polymorphic autoimmune syndrome, such as lupus, with a single and unique peptide, provided it is used at early stages, upstream of the cascade of immune events. With this information in mind, appropriate peptides and small molecules that selectively target the endo-lysosomal route, lysosomes, and MIIC compartment could be poised to be tools of choice to treat patients with efficacy and a minimum of safety risks and deleterious side effects.

## Conflict of Interest Statement

The authors declare that the research was conducted in the absence of any commercial or financial relationships that could be construed as a potential conflict of interest.
